# Human Cells Require Non-stop Ribosome Rescue Activity in Mitochondria

**DOI:** 10.1371/journal.pgen.1005964

**Published:** 2016-03-30

**Authors:** Heather A. Feaga, Michael D. Quickel, Pamela A. Hankey-Giblin, Kenneth C. Keiler

**Affiliations:** 1 Department of Biochemistry and Molecular Biology, The Pennsylvania State University, University Park, Pennsylvania, United States of America; 2 Department of Veterinary and Biomedical Science, The Pennsylvania State University, University Park, Pennsylvania, United States of America; University of California Santa Barbara, UNITED STATES

## Abstract

Bacteria use *trans*-translation and the alternative rescue factors ArfA (P36675) and ArfB (Q9A8Y3) to hydrolyze peptidyl-tRNA on ribosomes that stall near the 3' end of an mRNA during protein synthesis. The eukaryotic protein ICT1 (Q14197) is homologous to ArfB. In vitro ribosome rescue assays of human ICT1 and *Caulobacter crescentus* ArfB showed that these proteins have the same activity and substrate specificity. Both ArfB and ICT1 hydrolyze peptidyl-tRNA on nonstop ribosomes or ribosomes stalled with ≤6 nucleotides extending past the A site, but are unable to hydrolyze peptidyl-tRNA when the mRNA extends ≥14 nucleotides past the A site. ICT1 provided sufficient ribosome rescue activity to support viability in *C*. *crescentus* cells that lacked both *trans*-translation and ArfB. Likewise, expression of ArfB protected human cells from death when ICT1 was silenced with siRNA. These data indicate that ArfB and ICT1 are functionally interchangeable, and demonstrate that ICT1 is a ribosome rescue factor. Because ICT1 is essential in human cells, these results suggest that ribosome rescue activity in mitochondria is required in humans.

## Author Summary

Ribosomes can stall during protein synthesis on truncated or damaged mRNAs that lack a stop codon. In bacteria, these “non-stop” ribosomes are rescued by *trans*-translation or by an alternative rescue factor, ArfA or ArfB. Most eukaryotes do not have *trans*-translation, but mammals have a homolog of ArfB named ICT1. ICT1 is targeted to mitochondria, and is essential in human cells. Here, we show that human ICT1 and ArfB from the bacterium *Caulobacter crescentus* have the same biochemical activity and specificity. We also demonstrate that ICT1 and ArfB are functionally interchangeable in both bacteria and human cells. Collectively, this work demonstrates a new essential function in human cells—rescue of mitochondrial non-stop translation complexes.

## Introduction

The presence of a stop codon at the end of an open reading frame signals that the nascent protein is complete. Decoding of the stop codon by a release factor results in peptidyl-tRNA hydrolysis, releasing the completed protein and allowing the ribosome to be recycled [1]. Specific contacts between the release factors and bases in the stop codon are required for efficient catalysis of peptidyl-tRNA hydrolysis [2]. This stop codon recognition is necessary to prevent release factors from acting at sense codons and prematurely terminating translation. However, ribosomes can sometimes translate to the end of an mRNA without terminating at an in-frame stop codon. Translation cannot terminate normally at these “non-stop” complexes, because there is no stop codon in the decoding center to promote release factor activity. Ribosomes must be rescued from non-stop complexes so they can be recycled for productive protein synthesis [3,4].

In bacteria, non-stop complexes are rescued primarily by *trans*-translation. During *trans*-translation, a small protein, SmpB (P0A832), and a specialized RNA, tmRNA (EG30100), recognize a non-stop complex and release the ribosome at a stop codon within tmRNA. *trans*-Translation also targets the nascent polypeptide and mRNA from the non-stop complex for degradation [3,5]. Genes encoding tmRNA or SmpB have been identified in >99.9% of sequenced bacterial genomes [4]. *trans*-Translation is essential in some bacteria [6–8], but other species can survive without *ssrA* (encoding tmRNA) and *smpB* [9–11]. Some species, such as *C*. *crescentus*, have a severe growth defect when *ssrA* is deleted [12]. In other species, such as *Escherichia coli*, there is a relatively mild phenotype [13]. Synthetic-lethal screens have identified two alternative rescue factors, ArfA and ArfB, that can rescue non-stop complexes in the absence of *trans*-translation [14–16]. ArfA, found in *E*. *coli* and closely related bacteria, allows the release factor RF-2 to hydrolyze peptidyl-tRNA on non-stop ribosomes [17–20]. ArfB, found in *C*. *crescentus* and species from many phyla, contains a catalytic domain similar to release factors but does not include domains required for stop codon recognition. ArfB catalyzes hydrolysis of peptidyl-tRNA on non-stop ribosomes [15,16,21]. The C-terminal tail of ArfB is important for its activity [22], and structural studies suggest that it binds in the empty mRNA channel of non-stop ribosomes, similar to the C-terminal tail of SmpB [23]. *arfA* is essential in *E*. *coli* Δ*ssrA* cells [14], and *arfB* is essential in *C*. *crescentus* Δ*ssrA* cells [21], indicating that these species require at least one ribosome rescue mechanism. These observations have led to the suggestion that ribosome rescue activity may be essential for most or all bacteria [5].

**Fig 1:**
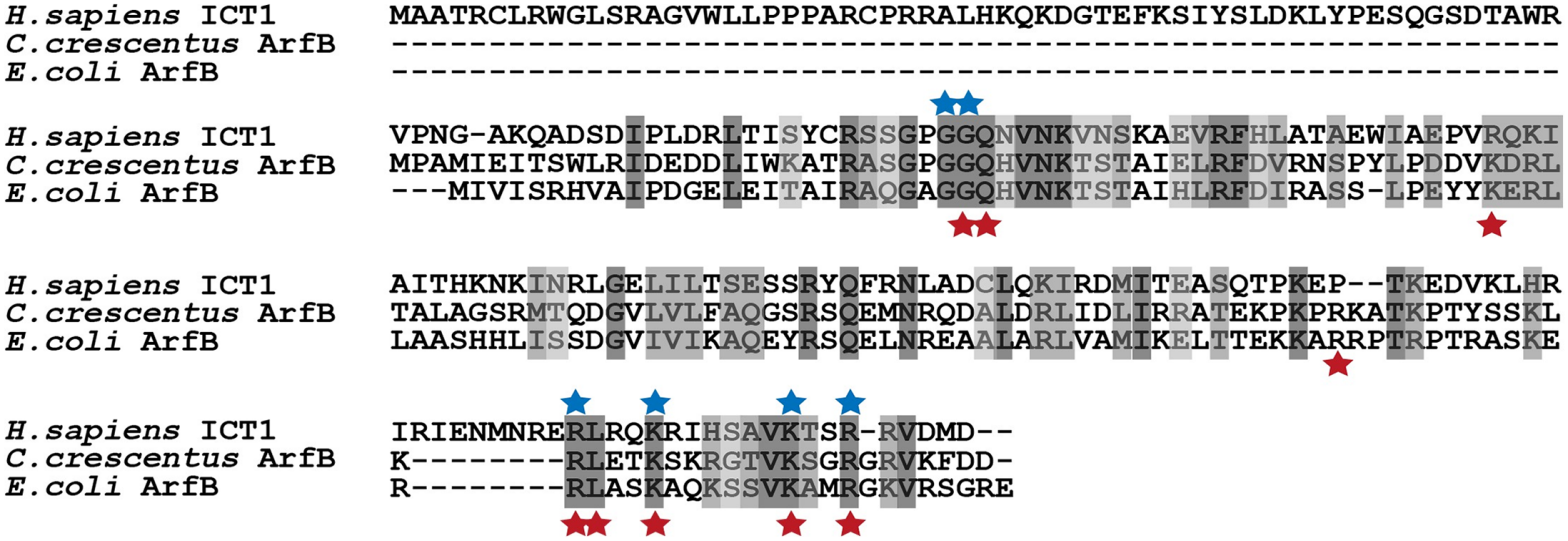
ICT1 and ArfB share conserved residues that are required for release activity. Clustal Omega alignment of human ICT1 and ArfB proteins from *E*. *COLI* and *C*. *CRESCENTUS*. Blue stars indicate residues required for ≥ 60% ICT1 peptidyl-tRNA hydrolysis activity on non-stop ribosomes [22]. Red stars indicate residues required for ≥ 60% *E*. *COLI* ArfB hydrolysis activity on non-stop ribosomes [22]. The N-terminal extension of ICT1 contains the mitochondrial localization signal. The remaining 143 C-terminal residues, thought to constitute the active portion of ICT1, share 26% sequence identity with *C*. *CRESCENTUS* ArfB.

Eukaryotes use Dom34/Pelota (P33309/Q9BRX2) and Hbs1 (P32769) to rescue ribosomes from non-stop mRNAs during translation in the cytoplasm [24,25], but factors required for this system are not present in mitochondria. Mammals have an ArfB homolog, ICT1, which is encoded in the nucleus and transported to mitochondria [26]. Knockdown experiments have demonstrated that ICT1 is essential in human cells [26,27], but conflicting models have been proposed to explain why ICT1 is essential [28–30]. Like ArfB, ICT1 can hydrolyze peptidyl-tRNA on *E*. *coli* non-stop ribosomes in vitro [22]. Because ICT1 can also hydrolyze peptidyl-tRNA on *E*. *coli* ribosomes assembled on short mRNAs with a stop or sense codon in the A site, it has been proposed to act as a general release factor that can terminate translation at any codon [26]. ICT1 has also been proposed to act on ribosomes stalled in the middle of an mRNA based on its ability to promote protein synthesis in reactions stalled by omission of a cognate release factor or tRNA [31]. Two mRNAs encoded in human mitochondria terminate with an AGA or AGG codon, so if ICT1 can act as non-specific release factor it might terminate translation of these messages. However, the sequence similarity between ICT1 and ArfB suggests that these factors are likely to have the same activity. ICT1 and ArfB share the conserved GGQ motif found in release factor catalytic domains, as well as residues in the C-terminal tail that are required for ArfB activity on non-stop ribosomes (Fig 1) [22,31]

Using a direct assay for peptidyl-tRNA hydrolysis in vitro, we find that the substrate specificity of ICT1 is almost identical to that of ArfB. Both factors catalyze peptidyl-tRNA hydrolysis on ribosomes stalled with no mRNA in the A site, or with mRNA extending a short distance past the A site, but have little activity on ribosomes stalled in the middle of an intact mRNA. In addition, we find that ArfB and ICT1 are interchangeable in vivo, both in *C*. *crescentus* and in human cells. These data indicate that ICT1 is a ribosome rescue factor and cannot terminate translation in the middle of mRNA, and suggest that mitochondrial ribosome rescue activity is essential in humans.

## Results

### Human ICT1 hydrolyzes peptidyl-tRNA on non-stop translation complexes

To evaluate the substrate specificity of ICT1, we used a gel-based assay to measure peptidyl-tRNA hydrolysis on *E*. *coli* ribosomes translating protein from mRNA. In these experiments, protein is produced using in vitro transcription-translation reactions and the components are separated on Bis-Tris gels that preserve the ester bond in peptidyl-tRNA. The fraction of protein that remains in the peptidyl-tRNA band indicates the extent of peptidyl-tRNA hydrolysis during the reaction [21].

**Fig 2:**
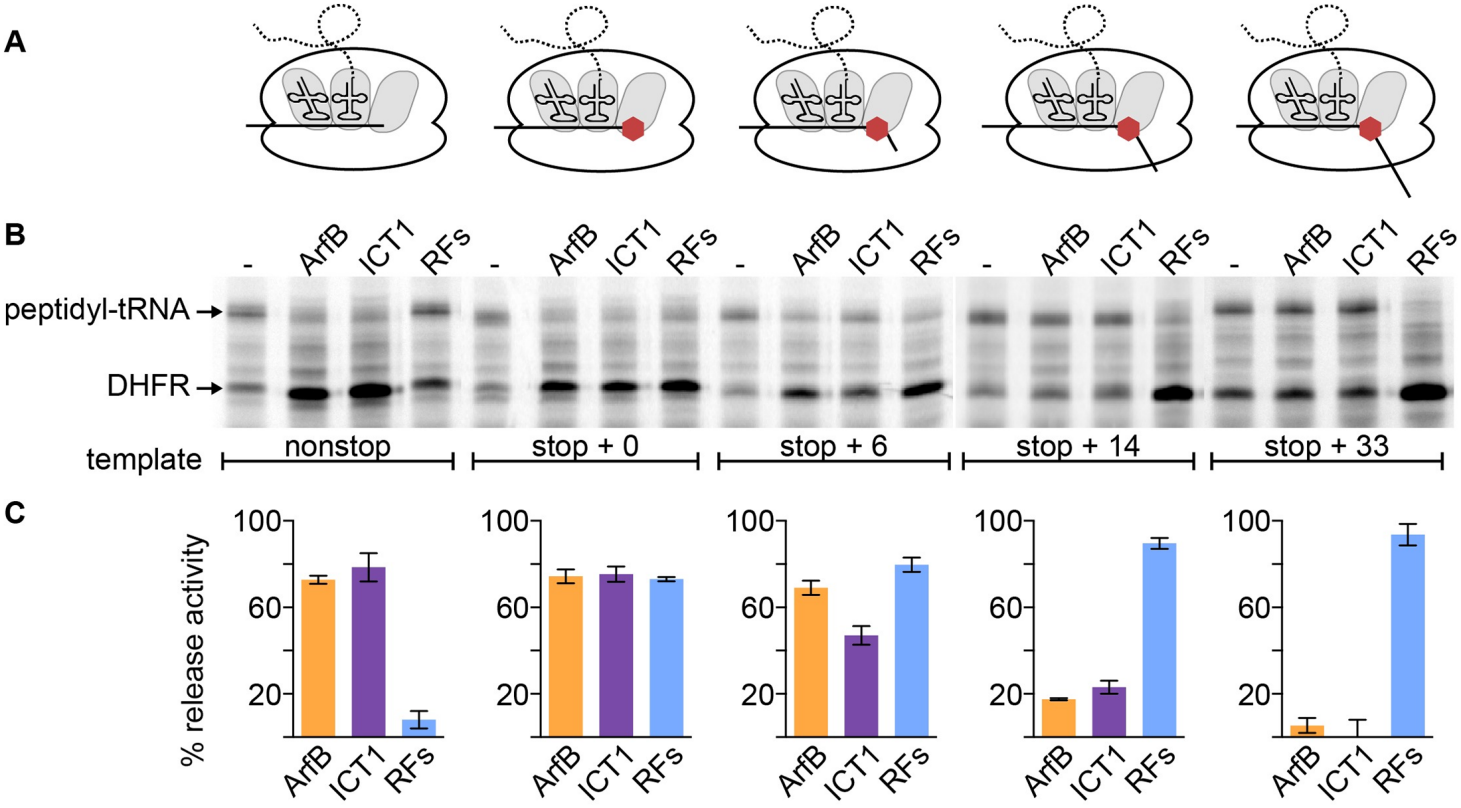
ICT1 and ArfB hydrolyze peptidyl-tRNA on ribosomes near the 3’ end of an mRNA. In vitro transcription/translation reactions were performed with template lacking a stop codon, or with template with 0, 6, 14, or 33 bases past the stop codon. (A) Cartoons depicting the expected result of translation in the absence of added rescue or release factors. (B) Representative autoradiograms of reactions resolved on Bis-Tris gels. (C) Column graphs show average release activity from ≥3 replicates with error bars indicating the standard deviation.

To confirm that ICT1 can hydrolyze peptidyl-tRNA on non-stop ribosomes in a manner similar to ArfB, a coupled transcription-translation system lacking RF1, RF2, and RF3 was used to express a *folA* gene (encoding DHFR) that lacked a stop codon. Consistent with previous observations of peptidyl-tRNA hydrolysis on non-stop ribosomes [15,16,21], addition of a release factor mixture containing RF-1, RF-2, and RF-3 to the reaction had little effect on the percentage of DHFR found in peptidyl-tRNA, but when ArfB was included 74 ± 1% of the DHFR was released (Fig 2). When ICT1 was included in the reaction, 78 ± 8% DHFR was released, indicating that ICT1 has a similar activity to ArfB on non-stop ribosomes.

To determine if ICT1 and ArfB can also release ribosomes stalled with mRNA extending into or past the A site, the peptidyl-tRNA hydrolysis assay was repeated with longer *folA* templates. A stop codon was added to the end of the non-stop template, and 0, 6, 14, or 33 nucleotides were added after the stop codon. Each template was designed to produce an mRNA with a stem-loop at the 3’ end to limit exonuclease activity during the reaction. Translation of these mRNAs will result in a ribosome stalled at the stop codon with peptidyl-tRNA in the P site. As expected, addition of release factors to reactions with any of these templates resulted in release of most of the peptidyl-tRNA (Fig 2). ICT1 and ArfB hydrolyzed peptidyl-tRNA as efficiently as release factors when the stop + 0 template was used. Substantial peptidyl-tRNA hydrolysis activity by ICT and ArfB was also observed with the stop + 6 template, but significantly less activity was observed when the template had a longer sequence past the stop codon (p < 0.001). Almost no hydrolysis was observed with ICT1 or ArfB on the stop + 33 template. These results indicate that ICT1 and ArfB can hydrolyze peptidyl-tRNA on ribosomes stalled near the 3’ end of an mRNA, and that a codon in the A site does not interfere with ribosome rescue. However, mRNAs that extend ≥ 14 bases past the A site substantially decrease activity of both ICT1 and ArfB.

#### Human ICT1 has ArfB activity in *C*. *crescentus*

**Fig 3:**
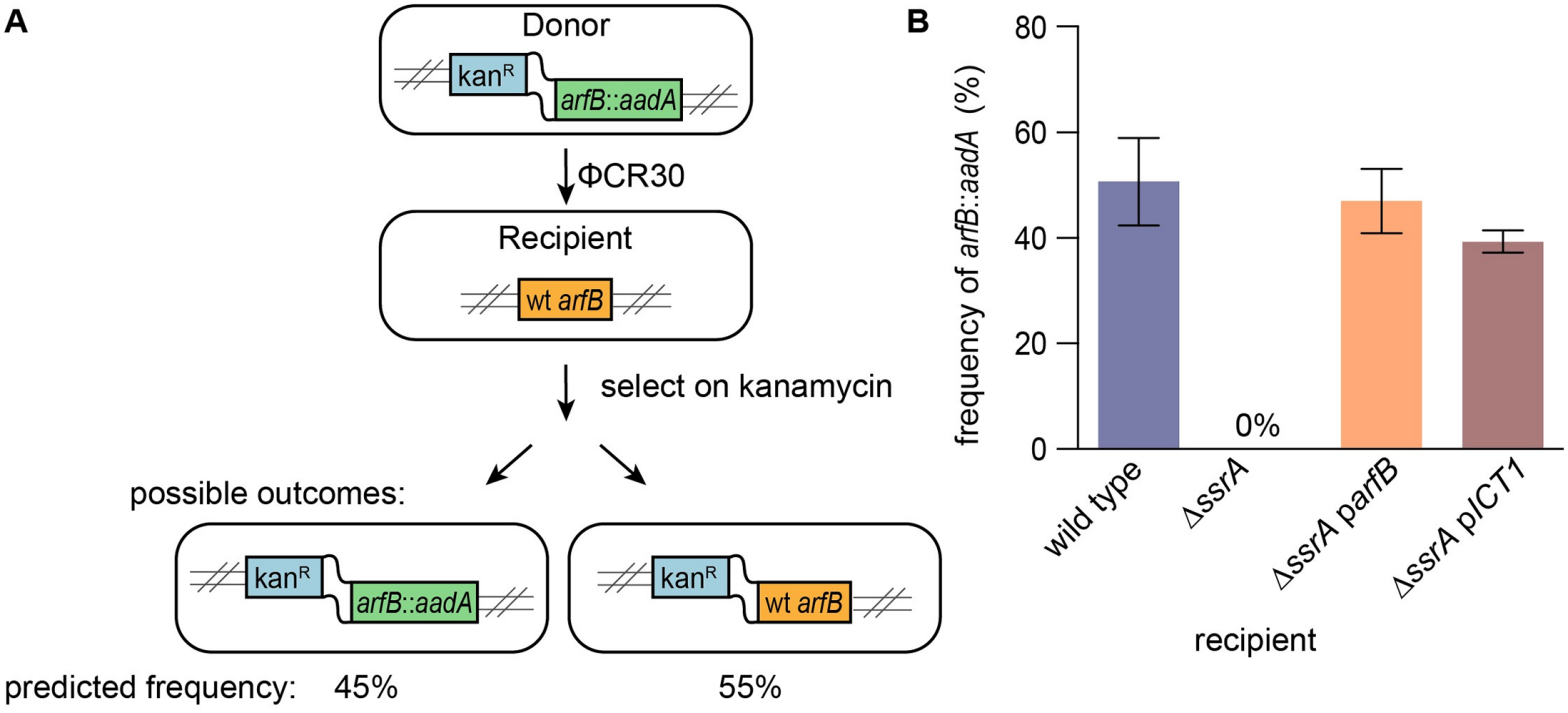
ICT1 ribosome release activity supports viability in *c*. *crescentus*. A co-transduction experiment was used to test whether ICT1 complements the synthetic lethal phenotype of deleting *ARFB* and *SSRA*. (A) Cartoon depicting the co-transduction experiment and predicted frequency of the outcomes if *ARFB* were not essential. (B) Column graph indicating the average co-transduction frequency from 3 independent experiments, with error bars indicating the standard deviation.

The activity of ICT1 and ArfB in vitro was similar enough to suggest that ICT1 might substitute for ArfB in *C*. *crescentus*. In a previous study, we used genetic linkage analysis to demonstrate that deletions of *ssrA* and *arfB* are synthetically lethal in *C*. *crescentus* [21]. A phage ΦCR30 lysate was prepared from strain KCK 428. In this strain, *arfB* is replaced with a copy of the *aadA* gene (conferring resistance to spectinomycin) and a copy of the *nptII* gene (conferring resistance to kanamycin) is inserted in the chromosome 22 kb from the *arfB* locus. The co-transduction frequency of the two antibiotic resistance markers could then be measured by infecting recipient cells with phage, selecting for transductants on kanamycin, and screening the kanamycin-resistant cells for spectinomycin resistance. Based on their relative locations on the chromosome, the predicted frequency with which the *aadA* and *nptII* genes should co-transduce when *arfB* is not essential is 45% [32]. Consistent with this prediction, when lysate is used to infect wild-type cells, *arfB*::*aadA* is recovered at a frequency of 51 ± 8% When this lysate was used to infect Δ*ssrA* cells, transductants with the *arfB* deletion were not recovered, indicating that *arfB* is essential in the absence of *ssrA*. To determine if ICT1 could provide ribosome rescue activity to support viability of *C*. *crescentus*, the co-transduction experiment was repeated using Δ*ssrA* cells that contained ICT1 (Fig 3). When the Δ*ssrA* strain expressed ICT1 from a plasmid, *arfB* could be deleted, as indicated by the co-transduction of *arfB*::*aadA* with the *nptII* gene. The co-transduction frequency of *aadA* and *nptII* into Δ*ssrA* cells expressing ArfB or ICT1 was not significantly different (p = 0.12), indicating that ICT1 can suppress the synthetic-lethal phenotype of deleting *arfB* and *ssrA*. In contrast, when Δ*ssrA* cells harboring empty plasmid were used as the recipient, none of >500 kanamycin-resistant transductions were resistant to spectinomycin (Fig 3).

#### ICT1 suppresses the growth defect in *C*. *crescentus* Δ*ssrA* cells

**Fig 4:**
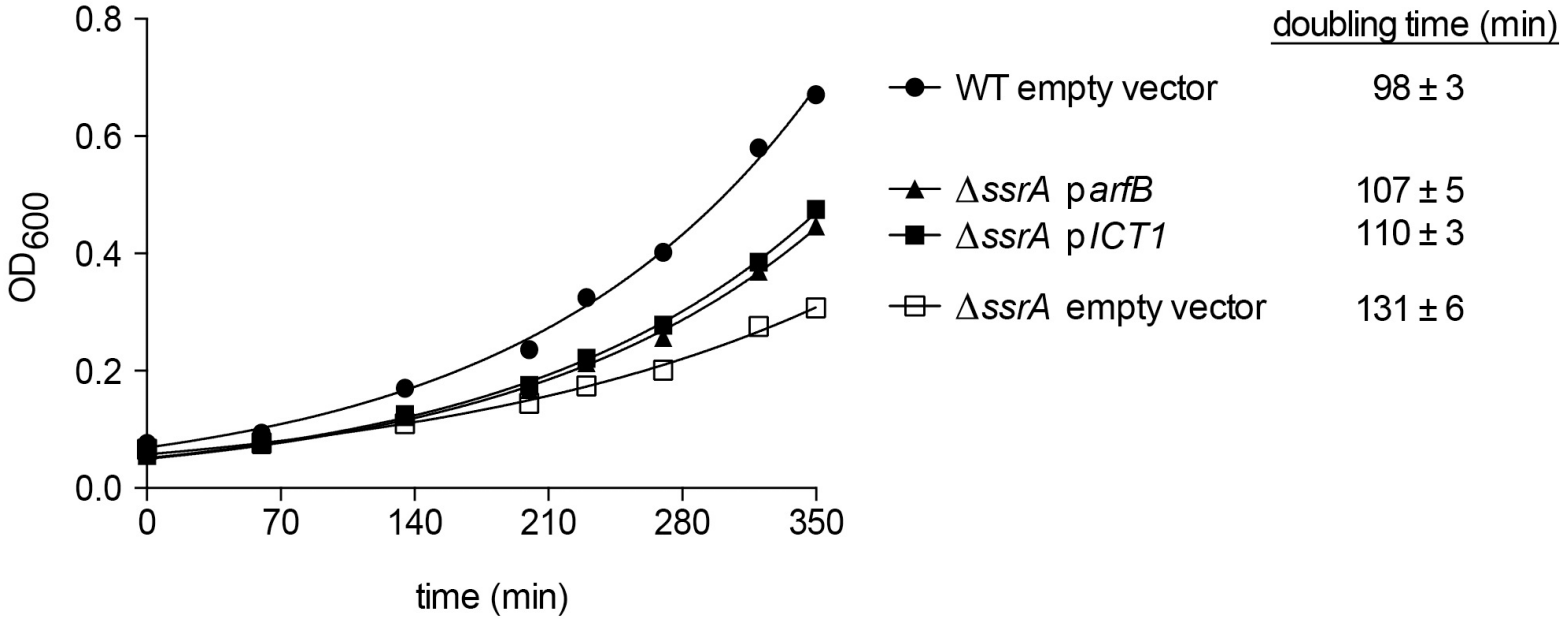
expression of ICT1 partially complements the growth defect of δ*ssra* cells. Growth of wild-type cells, Δ*SSRA* cells, or Δ*SSRA* cells expressing ArfB or ICT1 from a plasmid was monitored during exponential phase. The average doubling times (± standard deviation) from ≥3 experiments are shown.

Previous characterization of *trans*-translation and ArfB activity in *C*. *crescentus* showed that cells lacking *trans*-translation grow slowly [12], and over-expression of ArfB partially suppresses this growth defect [21]. To determine if ICT1 can perform the same function as ArfB in this assay, the growth rate of the Δ*ssrA* strain expressing ICT1 was measured and compared to control strains. Δ*ssrA* cells expressing ICT1 cells grew at a rate similar to Δ*ssrA* cells expressing ArfB, and significantly faster than Δ*ssrA* cells (p <0.01) (Fig 4). These results indicate that ICT1 activity supports the same rate of growth as ArfB activity, and suggest that human ICT1 is functionally interchangeable with ArfB in *C*. *crescentus*.

#### ArfB rescues human cells when ICT1 is silenced

**Fig 5:**
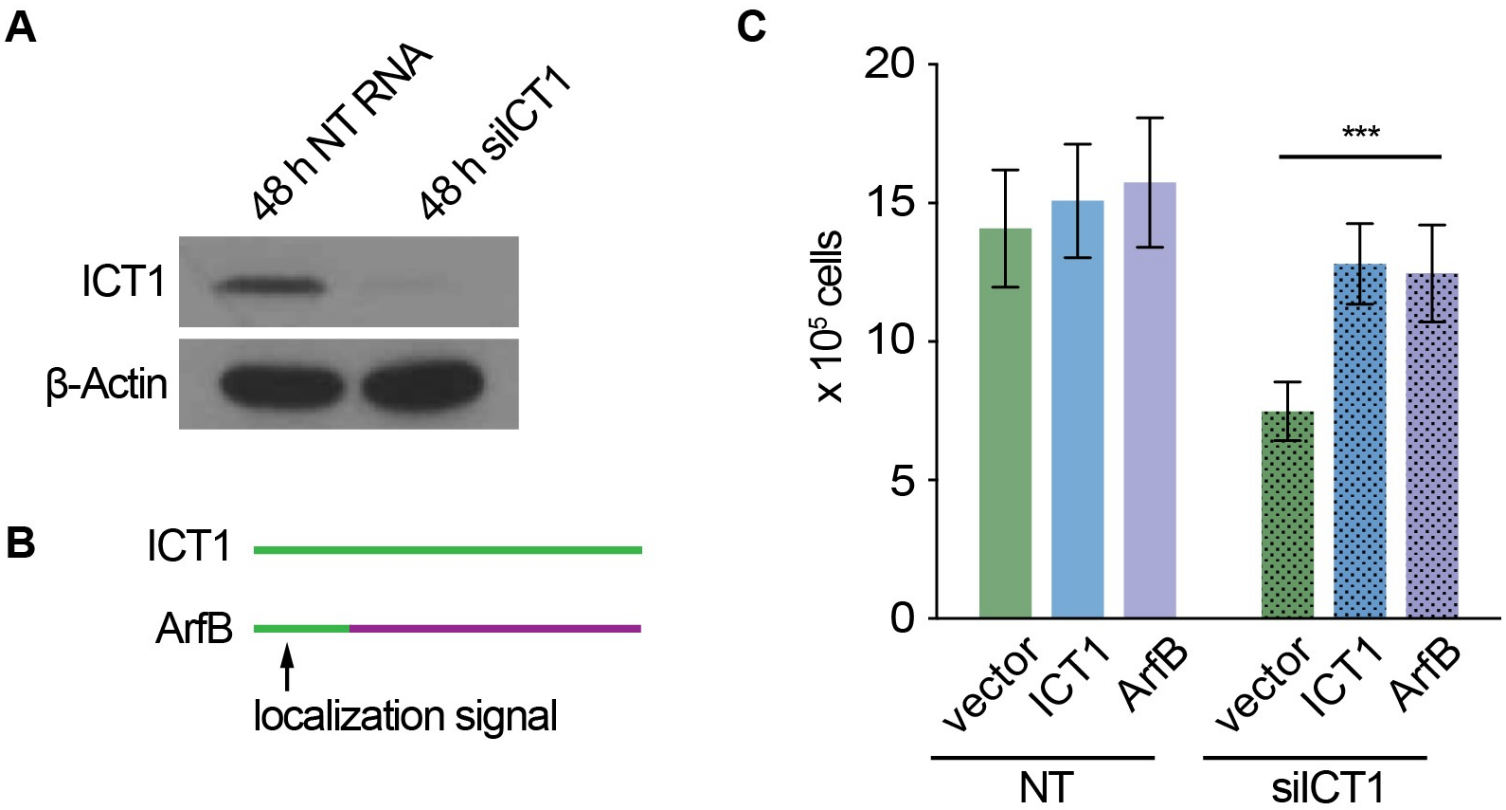
*c*. *crescentus* ArfB rescues human cells from ICT1 silencing. Viability of HEK293 cells expressing ICT1 or ArfB was determined after silencing endogenous ICT1. (A) Western blot showing depletion of ICT1 after silencing with siICT1. Non-targeting siRNA (siNT) was used as a negative control. (B) Schematic diagram showing ArfB with ICT1 localization signal that was used for rescue. (C) Column graphs showing average viable cell numbers from 5 independent experiments. Error bars indicate standard deviation. *** indicates p < 0.0001.

Mutant versions of ICT1 that lack the GGQ motif required for peptidyl-tRNA hydrolysis do not support viability in human cells when the endogenous ICT1 is silenced [26], indicating that ICT1-mediated peptidyl-tRNA hydrolysis is essential. If the essential activity of ICT1 is rescue of non-stop ribosomes, *C*. *crescentus* ArfB might be able to support viability in the absence of ICT1. To test this possibility, a gene encoding the N-terminal sequence of ICT1 containing the mitochondrial localization signal [33] fused to ArfB was constructed. HEK293 cells were transfected with a vector to express ArfB or ICT1, or with an empty vector. Eight hours later, these cells were transfected with siRNA that silenced ICT1 or with control siRNA, and after 6 days the number of living cells was determined. Consistent with previous results, ICT1 silencing decreased the number of viable cells, but cells expressing a version of ICT1 that was not silenced by the siRNA were not affected. Expression of ArfB prior to ICT1 silencing also prevented loss of viability. Cells expressing ArfB prior to ICT1 silencing grew to significantly higher numbers than cells transfected with vector alone (p < 0.0001) (Fig 5). There was no significant difference in viable cell number when cells were rescued with ArfB versus ICT1 (p = 0.75). These results indicate that ArfB can perform the essential function of ICT1 in human cells.

## Discussion

Rescue of ribosomes from non-stop translation complexes is a critical function for most bacteria, and the results presented here indicate that rescue of mitochondrial ribosomes is also essential in some eukaryotes. *C*. *crescentus* cells can survive without tmRNA and SmpB because they have ArfB to rescue ribosomes in the absence of *trans-*translation [21]. We have previously shown that *C*. *crescentus* ArfB can hydrolyze peptidyl-tRNA from non-stop ribosomes in vitro [21]. The data described here show that ArfB is also active on ribosomes stalled with a full codon in the A site or with 6 nucleotides past the A site, but longer mRNA extensions strongly inhibit ArfB activity. This substrate specificity is similar to that observed for tmRNA-SmpB [34–36], and indicates that ArfB is unlikely to interfere with translation elongation or with ribosomes paused during translation of full-length mRNAs. Instead, ArfB activity is consistent with a role in rescuing ribosomes that have translated to the 3’ end of an mRNA without terminating, ribosomes that have stalled after cleavage of the mRNA in the A site by a nuclease such as RelE [37], and stalled ribosomes that have had the 3’ portion of the mRNA removed by exonuclease activity [36,38].

Several lines of evidence demonstrate that human ICT1 is a ribosome rescue factor like ArfB, and not a non-specific release factor that can act on ribosomes stalled in the middle of an mRNA. First, the specificity of ICT1 in vitro for non-stop ribosomes or ribosomes with short mRNA extensions past the A site is similar to that of ArfB. Second, ICT1 can functionally replace ArfB in *C*. *crescentus*. Expression of ICT1 suppresses the synthetic lethality of deleting *ssrA* and *arfB*, and over-expression of ICT1 increases the growth rate in Δ*ssrA* cells to the same extent as over-expression of ArfB. Third, expression of ArfB in human cells suppresses the lethal effects of silencing ICT1, indicating that ArfB can functionally replace ICT1 in human cells. Finally, ICT1 would have little opportunity to act as a non-specific release factor during translation of intact transcripts in the mitochondria because mammalian mitochondrial transcripts are polyadenylated with ~50 nucleotides [39–44]. Based on the substrate specificity of ICT1 in vitro, this poly(A) tail would block ICT1 activity unless the mRNA was truncated, so ICT1 substrates for in vivo are likely to be non-stop complexes. Because ICT1 is essential in human cells [26,27], these results suggest that ribosome rescue in mitochondria is essential for human cell viability. The activity of ICT1 as a rescue factor and not a non-specific release factor would also explain why ICT1 does not interfere with translation elongation in mitochondria.

ICT1 substrate specificity has important implications for translation termination in mitochondria. Two human mitochondrial genes end in an AGG (*ND6*) or AGA (*COI*) codon. There are no cognate mitochondrial tRNAs for these codons and the mitochondrial release factor mtRF1a is unable terminate translation at AGA or AGG [45], so it is unclear how translation is terminated for these genes. ICT1 has been proposed to function as the termination factor at these codons based on analysis of activity in vitro on ribosomes stalled with an mRNA extending up to 14 nucleotides past the A site [30,31]. Our data show that ICT1 activity is greatly reduced on ribosomes stalled with an mRNA extending 14 nucleotides past the A site, and ICT1 activity is completely absent when the mRNA extends 33 nucleotides past the A site. Because the *COI* AGA codon is 72 nucleotides from the end of the transcript and the *ND6* AGG codon is 500–550 nucleotides from the end of the transcript [39,42], ICT1 should have no activity at these codons in either their unmodified or polyadenylated form. In addition, ICT1 can support viability in *C*. *crescentus*, so it cannot have an intrinsic ability to terminate translation at AGA or AGG because these codons encode arginine in bacteria. Likewise, ArfB does not recognize AGA or AGG in *C*. *crescentus*, so the ability of ArfB to replace ICT1 in human mitochondria suggests that termination at AGA or AGG in the middle of a transcript is not an essential function for ICT1. One possible mechanism for both ICT1 and ArfB to terminate translation at these codons is that stalling of the ribosomes leads to truncation of the mRNA 3’ of the ribosome, thereby producing a substrate for the rescue factors. A second possible mechanism for termination at AGA or AGG by ICT1 and ArfB would be that mitochondrial ribosomes might respond differently when they stall in the middle of an mRNA than do bacterial ribosomes. Mitochondrial ribosomes are descended from bacterial ribosomes, but are highly specialized for translating a small number of mRNAs encoded in the mitochondrial genome. The decoding center and peptidyl transfer center of mitochondrial ribosomes are very similar to bacterial ribosomes, but other architectural features are highly diverged [46]. For example, mammalian mitochondria have dramatically reduced rRNAs and lack 5S rRNA, but contain 36 proteins not found in bacteria and incorporate a tRNA as a structural component of the large subunit [47]. It is possible that this different architecture causes mitochondrial ribosomes to adopt a conformation that promotes rescue by ICT1 and ArfB when they are stalled in the middle of an mRNA. If ICT1 does not terminate translation at these codons, one of the other members of the mitochondrial RF family might perform this function.

Mitochondria descend from a progenitor of α-proteobacteria [48,49], the bacterial class that includes *C*. *crescentus*. Most α-proteobacterial species contain both *trans*-translation and ArfB, and some protist mitochondria encode *ssrA* and *smpB* [4], suggesting that the primordial mitochondrion had both ribosome rescue systems. Why did mitochondria keep ArfB and discard *trans*-translation, whereas almost all bacteria have kept *trans*-translation whether they have an alternative rescue factor or not? Mitochondria encode only 13 proteins, all of which are integral membrane proteins. Perhaps this limited proteome decreases the selective advantages of *trans*-translation, for example by enabling proteases to recognize incomplete versions of proteins without the tmRNA-encoded tag. Alternatively, the constraints of importing factors encoded in the nucleus might have favored ICT1 over tmRNA-SmpB, because ICT1 acts as a single protein. Interestingly, some plants encode an ArfB/ICT1 homolog with a chloroplast-targeting signal, (Q84JF2, e.g.) suggesting that ribosome rescue activity may be found in other organelles, and that the ArfB-type ribosome rescue may be generally favored in eukaryotic organelles.

## Materials and Methods

### Bacterial growth

Strains are described in Table 1. *C*. *crescentus* strains were grown at 30°C [50] in peptone-yeast extract (PYE) medium supplemented with tetracycline (2 μg/ml), streptomycin (50 μg/mL), spectinomycin (100 μg/mL), kanamycin (20 μg/mL), or xylose (0.3%) where appropriate. Growth was monitored by measuring optical density at 600 nm.

**Table 1:** Strains, plasmids, primers and RNAs used in this study.

Name	Description	Reference
**Strains**		
CB15N	Wild-type *C*. *crescentus*	[50]
CB15NΔ*ssrA*	In-frame deletion of *ssrA*	[12]
KCK426	*E*. *coli* BL21(DE3) carrying pET28*arfB*	[21]
KCK428	CB15N *arfB*::Ω with Kan marker 22.4 kb downstream	[21]
**Plasmids**		
pET28*arfB*	Used to express *C*. *crescentus* ArfB from T7 promoter	[21]
pET28*ICT1*	Used to express *H*. *sapiens* ICT1 from T7 promoter	This study
pMSCV*arfB*	pMSCVneo expressing ArfB with localization signal	This study
pMSCV*ICT1*	pMSCVneo expressing ICT1	This study
p*arfB*	encodes ArfB under the control of a xylose promoter	[21]
p*ICT1*	encodes ICT1 under the control of a xylose promoter	This study
**Primers**		
HAF_T7	CGAAATTAATACGACTCACTATAGGG	[21]
DHFR_NS_R	AAACCCCTCCGTTTAGAGAGGGGTTTTGCTAGTATCCGCCGCTCCAGAATCTCAAAGCAA	[21]
DHFR_stop_0	TTAACCCCTCCGTTTTAGAGAGGGGTTAATTGCTAGCCGCCGCTCCAGAATCTCAAAGCA	This study
DHFR_stop_6	AAACCCTTACTCCGTAGAGAGTAAGGGTTTTGCTAGCCGCCGCTCCAGAATCTCAAAGCA	This study
DHFR_stop_14	AAAAACCCCTCCGTTTAAGAGAGGGGTTTTGCTAGTATCCGCCGCTCCAGAATCTCAAAG	This study
DHFR_stop_33	AAAACCCCTCCGTTTAGAGAGGGGTTTTGCTAGTTACCGCCGCTCCAGAATCTCAAAGCA	This study
**RNAs**		
siNT	AGGUAGUGUAAUCGCCUUG dtdt	Eurofins Genomics
siICT1	GCCGCUAUCAGUUCCGGAA dtdt	[26]

### Plasmid construction

To construct pET28*ICT1* for expression and purification of mature ICT1, the coding sequence (codon-optimized for *E*. *coli*) ICT1 lacking the mitochondrial localization signal was purchased as a gBlock Gene Fragment (IDT) and inserted into pET28b by Gibson assembly [51]. Construction of p*arfB* (formerly p*CC1214*) has been described previously [26]. p*ICT1* was constructed by digesting pET28*ICT1* with NdeI and BamHI and ligating the resulting fragment into p*arfB* digested with the same enzymes. pMCSV*ICT1* was constructed by Gibson assembly of the human ICT1 sequence into the EcoRI and NotI sites of pMSCVneo. Alternative codons were selected for the region of ICT1 targeted by the siRNA to ensure that only endogenous ICT1 would be silenced. pMCSV*arfB* was similarly constructed by Gibson assembly of a gBlock Gene Fragment into pMSCVneo at the EcoRI and NotI sites. The *arfB* construct encodes the 62 residue N-terminal extension of human ICT1 to target it to mitochondria followed by the 142 residues of *C*. *crescentus* ArfB sequence codon-optimized for expression in human cells. The ArfB coding sequence was codon-optimized for expression in human cells.

### Purification of ICT1 and ArfB

Purification of *C*. *crescentus* ArfB has been described previously [21]. ICT1 was purified using a similar protocol. Strain KCK477 was grown to OD600 ~ 0.8 and expression was induced by addition of isopropyl-ß-D-thiogalactopyranoside (IPTG) to 1 mM. Cells were grown for 3 h at 37°C, harvested by centrifugation at 4°C, and resuspended in 30 ml lysis buffer (6 M guanidine hydrochloride, 20 mM sodium phosphate, 400 mM NaCl) [pH 7.8]. Cells were lysed by sonication and the lysates were cleared by centrifugation at 11,000 *g* for 30 min and applied to a column packed with 500 μl Ni-nitrilotriacetic acid (NTA) agarose (Qiagen) slurry equilibrated in DB buffer (8 M urea, 20 mM sodium phosphate 500 mM NaCl) [pH 7.8]. The column was washed 3X by rocking with 10 bed volumes DB buffer, washed 3X by rocking with 20 bed volumes DW buffer (8 M urea, 20 mM sodium phosphate, 500 mM NaCl) [pH 6.0], and washed 3X with 20 bed volumes of DW buffer [pH 5.3]. Protein was eluted in 1 ml fractions in elution buffer (8 M urea, 20 mM sodium phosphate, 500 mM NaCl) [pH 4.0]. Fractions containing ICT1 were dialyzed against ICT1 dialysis buffer (10 mM HEPES [pH 7.6], 150 mM NaCl). The 6 histidine tag was cleaved with Thrombin CleanCleave Kit (Sigma) at 4°C for 3 h according to the manufacturer’s instructions. Residual 6x-His tagged protein was removed by incubation with Ni-NTA agarose.

### Peptidyl-tRNA hydrolysis assays

Peptidyl-tRNA hydrolysis by ArfB, ICT1, and release factors was assayed using the PURExpressΔRF1,2,3 kit (New England Biolabs). Template used for in vitro transcription and translation was generated by PCR using the primers listed in Table 1. Each template was designed to produce an mRNA with a stem-loop at the 3’ end to limit exonuclease activity during the reaction. PURExpressΔRF1,2,3 kit components were mixed according to the manufacturer’s instructions and incubated with 200 nM ArfB, 200 nM ICT1, or 100 nM RF1, RF2 and RF3 for 1 h at 37°C. Anti-ssrA oligonucleotide was added to 5 μM to inhibit any *trans*-translation activity from tmRNA in the kit components. Samples were precipitated in 20 μl cold acetone, resuspended in loading buffer pH 6.5 (5 mM sodium bisulfite, 50 mM MOPS [morphonlinepropanesulfonic acid], 50 mM Tris, 1 μM EDTA, 0.1% SDS, 5% glycerol, 0.01% xylene cyanol, and 0.01% bromophenol blue), heated to 65°C for 5 minutes, and resolved on Bis-Tris gels with MOPS running buffer (250 mM MOPS, 250 mM Tris, 5 mM EDTA and 0.5% SDS).

### Phage transduction

ΦCR30 lysate was prepared from strain KCK428 as described previously [32]. The resulting lysate was used to transduce wild-type or Δ*ssrA* cells harboring p*ICT1*, p*arfB*, or empty vector. Overnights of each strain were grown in PYE supplemented with tetracycline and xylose to mid log phase. 25 μl ΦCR30 prepared from KCK428 was added and cultures were incubated at 30°C for 2.5 h with shaking. Cells were then plated on PYE with kanamycin and xylose to select for transductants. The resulting colonies were tested for spectinomycin and streptomycin resistance to determine the frequency with which *arfB*:*aadA* co-transduced.

### ICT1 silencing

Pre-annealed non-targeting control siNT, or targeting siICT1[26] RNAs were purchased from Eurofins MWG Operon. To demonstrate efficient knockdown, 7.5 × 10^5^ HEK293 cells were seeded in a 6-well plate and transfected once every 24 h with siNT and siICT1 as follows: a mixture containing 90 μl serum-free DMEM (Corning), 3.4 μl siRNA (20 μM stock), and 4 μl TransIT-siQUEST (Mirus Bio) was incubated at room temperature for 30 min and added to the cells. After 48 h, cells were lysed by addition of buffer containing 150 mM sodium chloride, 50 mM Tris [pH 8.0], 5 mM EDTA, 2 mM phenylmethylsulfonyl fluoride, 2 mM sodium orthovanadate, 10 mM sodium fluoride, and 1% Igepal TM CA-360 (USB). The efficacy of ICT1 silencing was then determined by western blot using polyclonal mouse anti-human ICT1 (Sigma).

### ICT1 rescue in HEK293 cells

HEK293 cells (ATCC) were seeded in 6-well plates at 5 × 10^4^ cells per well and allowed to adhere for 18 h. Cells were transfected by combining 1 μg pMSCV vector, pMSCV*ICT1*, or pMSCV*arfB*, 90 μl serum-free DMEM, and 3.8 μl TransIT-293 (Mirus Bio) and allowing the mixture to incubate for 30 min at room temperature. 8 h after transfecting with plasmid, cells were transfected with siNT or siICT1 according to the siRNA transfection protocol described in the previous section. Viable cell numbers were determined by 0.4% trypan blue staining of trypsinized cells 6 days after silencing.
